# Correction: Robrigado et al. Bridge Healing: A Pilot Project of a New Model to Prevent Repeat “Social Admit” Visits to the Emergency Department and Help Break the Cycle of Homelessness in Canada. *Int. J. Environ. Res. Public Health* 2023, *20,* 6845

**DOI:** 10.3390/ijerph21020194

**Published:** 2024-02-08

**Authors:** Matthew Robrigado, Igor Zorić, David A. Sleet, Louis Hugo Francescutti

**Affiliations:** 1 Faculty of Medicine and Dentistry, University of Alberta, Edmonton, AB T6G 1C9, Canada; zoric@ualberta.ca; 2 Rollins School of Public Health, Emory University, Atlanta, GA 30322, USA; davidasleet@gmail.com; 3 Bizzell US, New Carrollton, MD 20785, USA

There was an error in the original publication [1]. Section 2.5 “Preliminary Evaluation” was removed completely as the section contained information abstracted from an internal Alberta Health Services working document that was not meant to be shared publicly until the formal evaluation of Bridge Healing is completed.

The authors state that the scientific conclusions are unaffected. This correction was approved by the Academic Editor. The original publication has also been updated.
